# Identification of Sodium Transients Through Na_V_1.5 Channels as Regulators of Differentiation in Immortalized Dorsal Root Ganglia Neurons

**DOI:** 10.3389/fncel.2022.816325

**Published:** 2022-04-06

**Authors:** Antón L. Martínez, José Brea, Eduardo Domínguez, María J. Varela, Catarina Allegue, Raquel Cruz, Xavier Monroy, Manuel Merlos, Javier Burgueño, Ángel Carracedo, María Isabel Loza

**Affiliations:** ^1^BioFarma Research Group, Centro Singular de Investigación en Medicina Molecular y Enfermedades Crónicas (CIMUS), Universidade de Santiago de Compostela, Santiago de Compostela, Spain; ^2^Grupo de Medicina Xenómica, Centro de Investigación Biomédica en Red de Enfermedades Raras (CIBERER), Universidade de Santiago de Compostela, Santiago de Compostela, Spain; ^3^WeLab Barcelona, Parc Científic de Barcelona, Barcelona, Spain; ^4^Fundación Pública Galega de Medicina Xenómica, Instituto de Investigación Sanitaria de Santiago de Compostela (IDIS), SERGAS, Santiago de Compostela, Spain

**Keywords:** neuronal differentiation, immortalized DRG neurons, functional transcriptomics, electrophysiology, sodium currents

## Abstract

Neuronal differentiation is a complex process through which newborn neurons acquire the morphology of mature neurons and become excitable. We employed a combination of functional and transcriptomic approaches to deconvolute and identify key regulators of the differentiation process of a DRG neuron-derived cell line, and we focused our study on the Na_*V*_1.5 ion channel (encoded by *Scn5a*) as a channel involved in the acquisition of DRG neuronal features. Overexpression of *Scn5a* enhances the acquisition of neuronal phenotypic features and increases the KCl-elicited hyperexcitability response in a DRG-derived cell line. Moreover, pharmacologic inhibition of the Na_*V*_1.5 channel during differentiation hinders the acquisition of phenotypic features of neuronal cells and the hyperexcitability increase in response to changes in the extracellular medium ionic composition. Taken together, these data highlight the relevance of sodium transients in regulating the neuronal differentiation process in a DRG neuron-derived cell line.

## Introduction

Neuronal differentiation is a key process in neurodevelopment that consists of neurite growth or neuritogenesis, leading to synapse formation and neuronal connectivity, accompanied by other events such as the acquisition of excitability (Qian and Yang, 2009; Zamboni et al., 2018). Neuritogenesis is related to an increase in intracellular cAMP, which activates multiple protein kinases (Bennison et al., 2020). However, the mechanisms involved in this process have not been properly studied.

Defects in the neuronal differentiation process are related to a wide range of diseases, such as schizophrenia, Down syndrome, and autism spectrum disorder (Huo et al., 2018; Gandawijaya et al., 2021; Martínez et al., 2021). Furthermore, enhancing peripheral neuronal differentiation and neuritogenesis may represent a therapeutic strategy for nerve injury, which often results in neuronal damage leading to neuropathic pain (Navarro et al., 2007). Thus, a wider study of the mechanisms underlying those processes is required.

Primary neurons are the most representative models studying neuronal differentiation and neuritogenesis; however, their use has very significant drawbacks, such as the need to use animals for testing, the labor-intensive isolation procedures and the difficulty transfecting them (Doran et al., 2015). To overcome these issues, immortalized neurons have been employed for the study of neuronal differentiation, such as adrenal pheochromocytoma PC12 or neuroblastoma Neuro2a cell lines (Xiao and Liu, 2003; Cornell et al., 2016). Immortalized neuronal cells are able to proliferate, but when they are exposed to a differentiation medium, they stop cell division and develop phenotypic neuronal features (Haberberger et al., 2020).

Recently, we employed F11 cells, an immortalized neuronal cell line hybrid between murine neuroblastoma cells and rat dorsal root ganglia (DRG) neurons, to characterize the etiology and pharmacology of neuropathic pain. We established and validated a protocol to differentiate F11 cells employing forskolin and dibutyryl-cAMP, observing that this process induced the acquisition of neuronal features and increased their excitability (Martínez et al., 2019). F11 cell differentiation is a complex process mediated by activation of the cAMP response element-binding (CREB) transcription factor (Fioravanti et al., 2008; Isensee et al., 2014). This role for CREB in neuronal differentiation has also been observed in other neuronal types (Lonze and Ginty, 2002).

The employment of F11 cells in neuronal differentiation studies could have several advantages, such as F11 cells being peripheral neuron-derived cells, in contrast to most immortalized neuronal cell lines, and the DRG of adult individuals containing precursor cells that are able to proliferate and generate new neurons *in vitro* (Czaja et al., 2008; Gallaher et al., 2011). A wider understanding of the regulators of peripheral neuritogenesis and neuronal development could open a novel avenue for the enhancement of neural plasticity after peripheral nerve injury.

The transcriptome of undifferentiated F11 cells has been previously described (Yin et al., 2016); however, the transcriptome modifications induced by neuronal differentiation are not known. Thus, we aimed to establish the transcriptomic mechanisms involved in the differentiation process in F11 cells and to identify novel regulators of neuritogenesis and neuronal differentiation.

## Materials and Methods

### Reagents

KCl (131494; Panreac-AppliChem, Castellar del Vallès, Barcelona, Spain), CaCl_2_ (22320.298; VWR, Llinars del Vallès, Barcelona, Spain), and NaCl (S9888; Sigma–Aldrich, Madrid, Spain) were freshly weighed and dissolved before each assay. Thapsigargin (T9033; Sigma–Aldrich), PF-01247324 (PZ0274; Sigma-Aldrich), BIII 890CL (Boehringer Ingelheim, Sant Cugat del Vallès, Barcelona, Spain) and BIII-55CL (Boehringer Ingelheim) were diluted in DMSO (dimethyl sulfoxide; D8418; Sigma–Aldrich) at a stock concentration of 10 mM and stored at –20°C. Tetrodotoxin citrate (T-550; Alomone Labs, Jerusalem, Israel) was diluted in water at a stock concentration of 1 mM and stored at –20°C.

### Cell Culture and Differentiation

Mouse neuroblastoma/rat embryonic DRG neuron hybrid F11 cells (08062601; ECCAC, Salisbury, England, United Kingdom) were cultured in Dulbecco’s modified Eagle’s medium (DMEM) without sodium pyruvate (D5671; Sigma–Aldrich) supplemented with 10% (v/v) non-dialyzed fetal calf serum (FBS; F9665; Sigma–Aldrich), 2 mM glutamine (G7513; Sigma–Aldrich), 100 units/ml penicillin and 100 μg/ml streptomycin (P0781; Sigma–Aldrich) in a humidified atmosphere containing 5% carbon dioxide at 37°C.

Differentiation was achieved by exposing F11 cells in a humidified atmosphere with 5% carbon dioxide at 37°C to differentiation medium containing 1 mM dibutyryl-cAMP (N6,2′-O-dibutyryladenosine 3′,5′-cyclic monophosphate; sc-201567; Santa Cruz Biotechnologies, Heidelberg; Germany), 30 μM forskolin (sc-3562; Santa Cruz Biotechnologies), 0.5% dialyzed fetal calf serum (F0392; Sigma–Aldrich), 100 units/ml penicillin and 100 μg/ml streptomycin, and 2 mM glutamine in DMEM without sodium pyruvate for 72 h.

### Measurement of Calcium Transients

F11 cells were seeded into clear, flat-bottomed, black-walled, 384-well plates (781091; Greiner Bio-One, Frickenhausen, Baden-Württemberg, Germany) pretreated with 2 μg/ml laminin (L2020; Sigma–Aldrich) and 30 μg/ml poly-D-lysine (P6407; Sigma–Aldrich) at a density of 5,000 cells/well. Twenty-four hours later, the culture medium was replaced with differentiation medium in the corresponding wells. After 72 h, the medium was removed and replaced with 25 μL of fresh medium and incubated with 25 μL of FLIPR Calcium-6 dye (R8190; Molecular Devices, Sunnyvale, CA) diluted in HBSS (Hank’s Balanced Salt Solution; 14065-049; Gibco, Invitrogen, Carlsbad, CA, United States) containing 20 mM HEPES [(4-(2-hydroxyethyl)-1-piperazineethanesulfonic acid); H3375; Sigma–Aldrich] (pH 7.4) for 2 h at 37°C. Changes in intracellular calcium concentration [(Ca^2+^)_*i*_] were recorded using an FDSS7000EX Functional Drug Screen System (Hamamatsu Photonics, Cerdanyola del Vallès, Barcelona, Spain) by excitation of the calcium-sensitive fluorescent dye with a light source at 470–495 nm and emission in the 515–575 nm range. FDSS tips (A8687-62; Hamamatsu Photonics) were pretreated with a solution of 0.1% BSA (bovine serum albumin; 10775835001; Sigma–Aldrich) diluted in assay buffer. (Ca^2+^)_*i*_ responses were obtained as the difference between the maximum and the minimum fluorescence values by considering the peak elicited by KCl.

Store-operated calcium entry (SOCE) measurements were performed as previously described (Srivats et al., 2016). Briefly, F11 cells were seeded, differentiated and transfected as described above. FLIPR calcium-6 dye was dissolved in calcium-free HBSS (HEPES-buffered saline) in which Ca^2+^ was omitted and 1 mM BAPTA [1,2-bis(o-aminophenoxy) ethane-N,N,N′,N′-tetraacetic acid; A4926; Sigma–Aldrich] was added. After incubation for 2 h at 37°C, fluorescence was measured in an FDSS7000EX Functional Drug Screen System. Fluorescence was calibrated to calcium concentration [(Ca^2+^)] by following the formula:


$$\left[{\mathrm{Ca}}^{2+}\right]=K_{D}\times \left(\frac{F-F_{\min}}{F_{\max}-F}\right);$$

where *K*_*D*_ = 320 nM, *F* is the measured fluorescence, *F*_*max*_ is the fluorescence value determined after the addition of 0.1% Triton X-100 (101556683; Sigma–Aldrich) with 10 mM CaCl_2_ in HBS, and *F*_*min*_ is the fluorescence value determined after the addition of 0.1% Triton X-100 with 10 mM BAPTA in HBSS.

### Measurement of Intracellular Sodium Concentration

F11 cells were seeded into clear-bottom 96-well plates (6005558; Perkin-Elmer, Tres Cantos, Madrid, Spain) previously treated with 30 μg/ml poly-D-lysine at a density of 7,500 cells per well and differentiated as previously described. The Na^+^-specific fluorescent dye sodium-binding benzofuran isophthalate acetoxymethyl ester (SBFI-AM) (ab142800; Abcam, Cambridge, United Kingdom) was reconstituted with DMSO and diluted in HBSS containing 0.02% Pluronic F127 (P-3,000 MP; Thermo Fisher Scientific, Alcobendas, Madrid, Spain) to a final concentration of 7 μM according to a previously described protocol (Iamshanova et al., 2016). Cells were washed with HBSS, and 50 μL of SBFI solution was added per well. An Operetta High-Content Imaging System (Perkin-Elmer) was used to acquire fluorescence signals (10 fields per well, 40 × WD objective) taking four images, one with an excitation wavelength of 340 nm and an emission wavelength of 540 nm and the other with an excitation wavelength of 380 nm and an emission wavelength of 540 nm at 0 h and at 4 h after the addition of SBFI. Digital phase contrast images were also acquired to delimit the outline of the cells. Harmony High Content Imaging and Analysis Software (Perkin-Elmer) was used to determine the fluorescence intensity inside the cells. The output was calculated using the following formula:


Response=F3404/F3804F3400/F3800;

where *F*_340_4__ is the intracellular fluorescence emission of cells exposed to light with a wavelength of 340 nm at 4 h, *F*_380_4__ is the intracellular fluorescence emission of cells exposed to light with a wavelength of 380 nm at 4 h, *F*_340_0__ is the intracellular fluorescence emission of cells exposed to light with a wavelength of 340 nm at 0 h, and *F*_380_0__ is the intracellular fluorescence emission of cells exposed to light with a wavelength of 380 nm at 0 h.

### Microscopy Assays

Cells were seeded in 50 μL of culture medium at a density of 7,500 cells per well into clear-bottom 96-well plates previously treated with 30 μg/ml poly-D-lysine. After 24 h, the culture medium was replaced with differentiation medium as appropriate. Seventy-two hours after medium replacement, F11 cells were fixed in 4% paraformaldehyde (sc-281692; Santa Cruz BT) in PBS solution at 4°C for 20 min. Cells were then washed twice with HBSS before being permeabilized with blocking buffer containing 5% BSA and 0.1% Triton X-100 (T8787; Sigma–Aldrich) in HBSS for 30 min at room temperature. Then, the cells were stained with Alexa 488 dye-conjugated anti-β-tubulin mouse antibody (558605; Becton, Dickinson and Company Biosciences, San Agustín de Guadalix, Madrid, Spain) diluted 1:500 and 2.5 μM nuclear stain DRAQ5 (108410; Abcam) for 1 h at room temperature. An Operetta High-Content Imaging System was used to acquire bright field images and fluorescence signals (12 fields per well, 20 × WD objective). Image analysis was performed using Harmony High Content Imaging and Analysis Software considering the maximum neurite length in each well.

Time-lapse videos were recorded by seeding 3,500 F11 cells per well into clear-bottom 96-well plates previously treated with 30 μg/ml poly-D-lysine. After 24 h, the culture medium was replaced with differentiation medium as appropriate, and the plate was placed in the Operetta High-Content Imaging System in a humidified atmosphere containing 5% carbon dioxide at 37°C. Images were acquired employing a 10X objective in digital phase-contrast mode every 30 min for 72 h. The video was edited using Wondershare Filmora 9.3.5 software for Mac (Wondershare, Shenzhen, China). Image analysis was performed using Harmony High Content Imaging and Analysis Software considering the mean neurite length in each well. The mean neurite length of the cells in each well was quantified, and the mean neurite length of F11 cells after 72 h of exposure to differentiation medium was normalized to 100%.

Na_*V*_1.5 channel staining was performed in fixed and permeabilized cells as previously described. Cells were exposed to a solution of an anti-Na_*V*_1.5 rabbit antibody (Asc-005; Alomone Labs) diluted 1:200 in DMEM at 4°C. Eighteen hours later, the cells were washed and incubated with a solution of a goat anti-rabbit antibody conjugated with Alexa Fluor 647 (150083; Abcam) diluted 1:200 and 2.5 μg/ml Hoechst 33342 (H3570; Sigma–Aldrich) for 1 h at room temperature. After washing with HBSS twice, a High Content Imaging System Operetta was used to acquire bright field images and fluorescence signals (40 × WD objective, 24 fields per well). Image analysis was performed using Harmony High Content Imaging and Analysis Software by measuring the intensity of Alexa Fluor 647 fluorescence in the cytoplasm of the cells.

### Membrane Potential Monitoring With FluoVolt

F11 cells were seeded at a density of 2,500 cells/well into clear, flat-bottomed, black-walled, 384-well plates pretreated with 30 μg/ml poly-D-lysine. Twenty-four hours later, the culture medium was replaced with differentiation medium. Seventy-two hours later, cells were loaded in HBSS with HEPES (pH = 7.4) supplemented with the dye (1:1,000) and with the PowerLoad Concentrate (1:100), both included in the FluoVolt Membrane Potential Kit (F10488; Thermo Fisher Scientific), for 30 min at 37°C. Then, the background suppressor (Neuro Background Suppressor) diluted 1:20 in HBSS with HEPES was added to the wells. Changes in membrane potential after the addition of KCl were measured with an FDSS7000EX Functional Drug Screen System with a light source at 470–495 nm and emission in the 515–575 nm range. FDSS tips were pretreated with a solution of 0.1% BSA diluted in assay buffer.

### RNA Sequencing

Twenty million F11 cells per dish were seeded in Corning square bioassay dishes (CLS43110; Sigma–Aldrich), and 24 h later, the culture medium was replaced with differentiation medium. After 72 h, total RNA from F11 cells was extracted and purified employing the RNeasy Mini Kit (Qiagen, Hilden, Germany). RNA quantity, integrity and quality were estimated using a 2100 Agilent Bioanalyser (Agilent Technologies, Las Rozas, Madrid, Spain) to calculate RIN (RNA integrity number). The RIN value of all the samples was greater than 8. A NextSeq 500 platform (Illumina, San Diego, CA, United States) was employed to sequence the transcriptome of F11 cells. Three RNA sequencing (RNA-Seq) experiments were performed using independent samples.

### Bioinformatics Analysis of RNA-Seq Data

Reads were individually mapped to both the rat genome RGSC Rnor_5.0/rn5 and the mouse genome assembly NCBI37/mm9. First, the quality of the generated data was evaluated using FastQC and Prinseq (Babraham Bioinformatics, Cambridge, United Kingdom). A total of 440 M high-quality reads were obtained (91% of total reads). Next, detection of duplicated sequences was performed using Picard tools (Picard tools, Cambridge, MA, United States) and aligned with the specific gene sequence using the Spliced Transcripts Alignment to a Reference (STAR) algorithm. Quality control of the generated data was performed using RNA-SeQC. Gene expression quantification was performed using Hypergeometric Optimization of Motif EnRichment (HOMER) software. Differential expression analysis was performed using the *edgeR* R package (Robinson et al., 2009). Sequences were previously filtered by selecting only genes present in more than two samples with a count value greater than 1 cpm. Multidimensional scaling was additionally performed to explore the overall differences between groups. A quasi-likelihood *F*-test was used to test for differential expression using a negative binomial generalized linear model. Subsequently, the top differentially expressed genes (FDR adjusted *p*-value < 0.05) were employed to perform a biological interpretation analysis of the differential expression between categories using IPA^®^ (Ingenuity Pathway Analysis) software (Qiagen).

Discriminant analysis of principal components (DAPC) was performed using the adegenet package for R software (Jombart et al., 2010). Gene counts in each group were previously normalized and logarithmically transformed using the DESeq2 library (Love et al., 2014). The analysis was performed on the total number of genes previously filtered, and the group of genes with a higher contribution to the discriminant functions was identified.

### Real Time Reverse Polymerase Chain Reaction (RT–qPCR)

RNA for RT–qPCR was acquired using the same methodology as for RNA sequencing. RT–qPCR was performed using 5 ng of RNA with an EXPRESS One-Step Superscript qRT–PCR kit (11781200; Thermo Fisher Scientific). Gene expression was determined using TaqMan assays on a QuantStudio 12K Flex reader (Life Technologies, Carlsbad, CA, United States). Gene-specific probes and primers were purchased from Applied Biosystems (Foster City, CA, United States). The following TaqMan gene expression assays were employed: *Scn5a* (Mm01342518_m1), *Shc2* (Mm01171467_m1) and *Neurog2* (Mm00437603_g1). All templates were analyzed in triplicate in three independent experiments, and relative expression levels were calculated for each sample after normalization against 36b4 as reference gene, using the ΔΔC_*t*_ method for comparing relative fold expression differences, as previously described (Livat and Schmittgen, 2001).

### Plasmid Transfection

The *SCN5A* plasmid (OHu18270; GenScript, Leiden, Netherlands) and pcDNA3 empty vector (as a gift from Marian Castro) were purified using a Nucleobond Xtra Midi EF Kit (740420; Macherey-Nagel GmBH, Düren; Germany).

Forward transfection was performed using 0.0768 μg of DNA and 0.2304 μL of Lipofectamine 3000 (L3000001; Thermo Fisher Scientific) per well (ratio 1:3). Cells were seeded in culture medium into the corresponding plates for each experiment, and 24 h later, the culture medium was replaced with DNA and Lipofectamine mix in Opti-MEM (11058021; Gibco). After 4 h, the transfection reagents were replaced with fresh culture medium for 72 h. Transfection efficiency was confirmed by parallel transfection with a construct coding for GFP protein, and overexpression was also confirmed by RT–qPCR.

### Data Analysis

Data analysis was performed using GraphPad Prism 6.0 software (GraphPad Software, La Jolla, CA, United States). Data were compared using Student’s *t*-test or ANOVA test followed by Dunett’s *post hoc* analysis for multiple comparisons. Differences were considered statistically significant at a *p*-value < 0.05.

## Results

### Differentiation of F11 Cells Induces the Acquisition of Neuronal Phenotypic Features With Changes in Intracellular Ion Homeostasis

**FIGURE 1:**
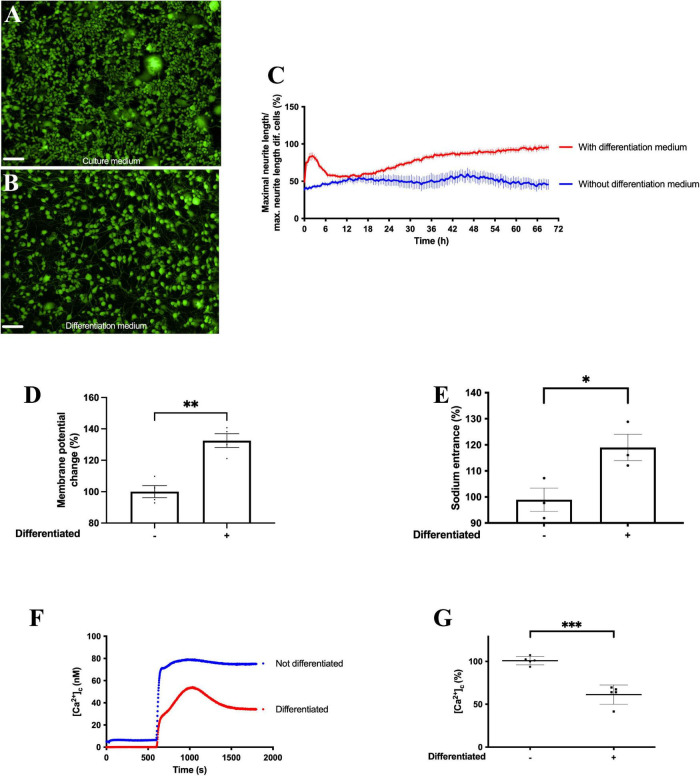
Differentiation of F11 cells induces increased neurite length and enhanced changes in intracellular sodium homeostasis and cell membrane potential. Representative images of F11 cells **(A)** grown in culture medium and **(B)** exposed to differentiation medium for 72 h. Images are representative of two assays (*n* = 2) with 11 replicates per condition, 10X. Scale bar = 100 μm. **(C)** Variations in neurite length after 3 days of exposure to differentiation medium and to culture medium. Values shown for **(C)** are the means ± SEM of two independent assays (*n* = 2) with 11 replicates per measurement. **(D)** Change in membrane potential measured with FluoVolt elicited by 30 mM KCl before and after differentiation. Values shown are the means ± SD of four independent assays (*n* = 4) with three replicates per measurement. ^**^p < 0.01 (Student’s *t-*test). **(E)** Intracellular sodium concentrations in F11 cells before and after differentiation measured with SBFI. Values shown are the means ± SEM of three independent assays (*n* = 3) with four replicates per measurement. *p < 0.05 (Student’s *t-*test). **(F)** Calcium transients evoked by calcium restoration after exposure to 1 μM thapsigargin in non-differentiated and differentiated F11 cells and **(G)** summary results of responses to calcium restoration 16.6 min after the addition of thapsigargin in non-differentiated and differentiated F11 cells. Values shown for **(F)** are the means of one representative assay of five independent experiments with 12 replicates per measurement. Values shown for **(G)** are the means ± SD of five independent assays (*n* = 5) with 12 replicates per measurement. ^***^p < 0.001 (Student’s *t-*test).

Exposure of F11 cells to differentiation medium for 72 h induced the acquisition of neuronal phenotypic characteristics, such as an increase in neurite length (Figures 1A–C, Supplementary Figure 1, and Supplementary Video 1). F11 cell differentiation also induced a significant increase in KCl-induced membrane depolarization (Figure 1D) (*p* < 0.01, Student’s *t*-test) (Supplementary Figure 2), a significant increase in intracellular sodium concentration (Figure 1E) (*p* < 0.05, Student’s *t*-test) and a significant reduction in SOCE (store-operated calcium entrance) (Figures 1F,G) (*p* < 0.001, Student’s *t*-test).

### Differentiation of F11 Cells Upregulates the Transcription of Genes Related to Membrane Potential and Downregulates Genes Related to Cell Proliferation

**FIGURE 2:**
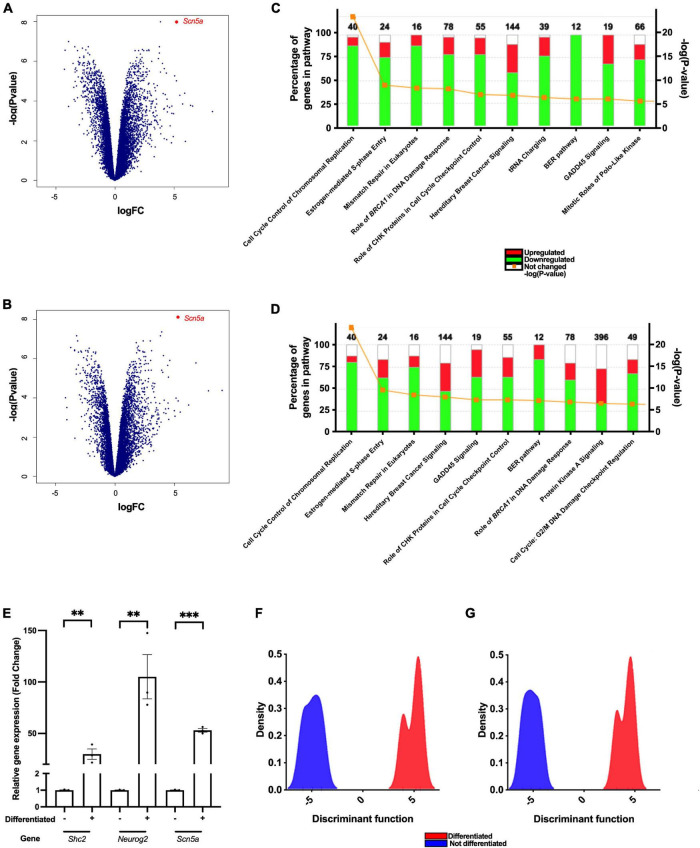
Differentiation of F11 cells enhances the transcription of genes related to the regulation of membrane potential, neuritogenesis and neuronal differentiation. Cloud plot depicting changes in the expression of **(A)** mouse and **(B)** rat genes after differentiation of F11 cells. Points represent the mean of the values obtained from three independent RNA-Seq experiments performed using different samples. Changes observed in genes belonging to **(C)** mouse and **(D)** rat signaling pathways after differentiation of F11 cells. **(E)** Comparative quantification of the *Scn5a*, *Shc2* and *Neurog2* gene expression in non-differentiated and differentiated F11 cells employing RT–qPCR. Values shown are the means ± SD of three independent assays (*n* = 3) with three replicates per measurement. ^***^p < 0.001; ^**^p < 0.01 (Student’s *t-*test). Discriminant analysis of principal components of **(F)** mouse and **(G)** rat genes comparing non-differentiated and differentiated F11 cells.

To identify the pathways and genes involved in F11 cell differentiation, we compared variations in the transcriptome of this cell line during the differentiation process. We observed increased expression of 7,073 mouse genes and decreased expression of 6,807 mouse genes (Figure 2A), and increased expression of 6,178 rat genes and decreased expression of 5,113 rat genes (Figure 2B). Among the upregulated genes, we observed that differentiation elicited an increase in the transcription of genes related to the regulation of membrane potential, such as *Scn5a* or *Scn10a*, and genes related to neuronal development, such as *Neurog2* or *Shc2* (Table 1).

**TABLE 1:** Top 25 significantly upregulated mouse and rat genes in response to neuronal differentiation of F11 cells.

Mouse			Rat		
Position	Expr. log ratio	Symbol	Position	Expr. log ratio	Symbol
1	8.24	*Adipoq*	1	9.01	*Adipoq*
2	6.59	*Slpi*	2	7.88	*Scn10a*
3	6.39	*Arid3c*	3	6.82	*Zmat4*
4	5.95	*Gm20594*	4	6.19	*Ackr3*
5	5.89	*Zmat4*	5	5.51	*Epha3*
6	5.72	*Ackr3*	6	5.32	*Igf2*
7	5.50	*Igf2*	7	5.29	*Scn5a*
8	5.21	*Scn5a*	8	5.13	*Nppc*
9	5.21	*Shc2*	9	5.05	*Otof*
10	5.05	*Neurog2*	10	5.00	*Shc2*
11	4.96	*Otof*	11	4.60	*Vip*
12	4.93	*Snora81*	12	4.54	*H19*
13	4.83	*Loxl1*	13	4.51	*Aass*
14	4.83	*Nppc*	14	4.49	*Mgst3*
15	4.80	*Snora23*	15	4.46	*Vdr*
16	4.60	*Scn10a*	16	4.44	*Fst*
17	4.60	*Tspoap1*	17	4.30	*Tmem184a*
18	4.43	*H19*	18	4.27	*Rspo1*
19	4.43	*Adgrg1*	19	4.14	*Padi3*
20	4.40	*Rspo1*	20	4.11	*Slc4a1*
21	4.39	*Vaultrc5*	21	4.08	*Pde3a*
22	4.36	*Fst*	22	4.02	*Lrp1b*
23	4.26	*Efcab6*	23	3.93	*Scara5*
24	4.17	*Hba1/Hba2*	24	3.93	*Kirrel2*
25	4.17	*Slc4a1*	25	3.89	*Lynx1*

*Data are shown as the mean of three independent experiments using different samples.*

In contrast, after F11 cell differentiation, there was a reduction in the transcription of genes involved in pathways related to cell cycle regulation (Figures 2C,D and Table 2).

**TABLE 2:** Top 25 significantly downregulated mouse and rat genes in response to neuronal differentiation of F11 cells.

Mouse			Rat		
Position	Expr. log ratio	Symbol	Position	Expr. log ratio	Symbol
13,880	–4.28	*Hist1h2ba*	11,291	–4.14	*Hsd11b2*
13,879	–4.19	*Hist1h2bb*	11,290	–4.04	*Hist1h2ba*
13,878	–3.92	*Taf7 l*	11,289	–4.02	*Myl7*
13,877	–3.91	*Hist1h1b*	11,288	–3.71	*Hist1h2bk*
13,876	–3.53	*Rrm2*	11,287	–3.39	*Hist1h1b*
13,875	–3.47	*Hist1h2aj*	11,286	–3.38	*Rrm2*
13,874	–3.43	*Hist1h2ab*	11,285	–3.36	*Pimreg*
13,873	–3.38	*Mcm5*	11,284	–3.25	*Pclaf*
13,872	–3.37	*Hist1h2al*	11,283	–3.23	*Trip13*
13,871	–3.37	*Pclaf*	11,282	–3.21	*Hist1h2bo*
13,870	–3.36	*Pimreg*	11,281	–3.13	*Mcm5*
13,869	–3.36	*Myb*	11,280	–3.06	*Cdc20*
13,868	–3.28	*Hist1h2bn*	11,279	–3.06	*Hist1h2ak*
13,867	–3.24	*Hist1h2ah*	11,278	–2.98	*Angpt2*
13,866	–3.20	*Trip13*	11,277	–2.93	*Tk1*
13,865	–3.16	*Ndp*	11,276	–2.92	*Mybl2*
13,864	–3.14	*Hist1h3 h*	11,275	–2.89	*Dscc1*
13,863	–3.10	*Hist1h2ac*	11,274	–2.85	*Mcm3*
13,862	–3.09	*Tk1*	11,273	–2.84	*Hist1h2bl*
13,861	–3.04	*Cdc20*	11,272	–2.84	*Hist1h2bn*
13,860	–3.03	*Hist1h2ad*	11,271	–2.80	*Hist1h2al*
13,859	–3.02	*Hist1h2bc*	11,270	–2.80	*Igf1*
13,858	–2.99	*Myl7*	11,269	–2.76	*Hist1h1a*
13,857	–2.97	*Mcm3*	11,268	–2.74	*Cdca8*
13,856	–2.93	*Hist1h2be*	11,267	–2.73	*Cd244*

*Data are shown as the mean of three independent experiments using different samples.*

### Increased Expression of Na_V_1.5 Voltage-Gated Sodium Channels Enhances the Acquisition of Neuronal Phenotypic Features in F11 Cells

We observed that *Scn5a*, which encodes Na_*V*_1.5 voltage-gated sodium channel, was overexpressed during F11 cell differentiation. The increased transcription of *Scn5a* in differentiated F11 cells compared to non-differentiated F11 cells was significant as assessed by RT–qPCR in independent assays (Figure 2E) (*p* < 0.001, Student’s *t*-test). The importance of this gene in differentiation was also shown in the DAPC of the RNA-Seq results (Figures 2F,G and Table 3). We also confirmed an increase in the expression of Na_*V*_1.5 channels in the cell membrane of differentiated F11 cells by immunofluorescence microscopy (Supplementary Figure 3) (*p* < 0.001, Student’s *t*-test).

**TABLE 3:** F11 mouse and rat genes with the highest contribution to distinguishing the neuronal differentiation functional transcriptome assessed by performing a discriminant analysis of principal components (DAPC).

Mouse				Rat			
Symbol	Contribution to DF	Log (exp)	–Log P	Symbol	Contribution to DF	Log (exp)	–Log P
*Igf2*	5.0⋅10^–3^	5.5	5.7	*Igf2*	5.9⋅10^–3^	5.3	5.1
*Snora23*	4.1⋅10^–3^	4.8	2.5	*Scn5a*	4.4⋅10^–3^	5.3	12
*Snora81*	3.9⋅10^–3^	4.9	2.9	*H19*	4.1⋅10^–3^	4.5	3.9
*Lars2*	3.8⋅10^–3^	2.2	0.6	*Hist1h2bk*	3.4⋅10^–3^	–3.7	9.6
*Hist1h2bb*	3.6⋅10^–3^	–4.2	9.6	*Hist1h1b*	3.4⋅10^–3^	–3.4	9.2
*Vaultrc5*	3.3⋅10^–3^	4.4	2.7	*Rrm2*	3.1⋅10^–3^	–3.4	9.3
*Hist1h1b*	3.2⋅10^–3^	–3.9	11	*Rn18s*	3.1⋅10^–3^	3.3	1.8
*Scn5a*	3.2⋅10^–3^	5.2	12	*Fst*	3.1⋅10^–3^	4.4	7.9
*H19*	3.1⋅10^–3^	4.4	4.5	*Hist1h2bo*	3.0⋅10^–3^	–3.2	11

**FIGURE 3:**
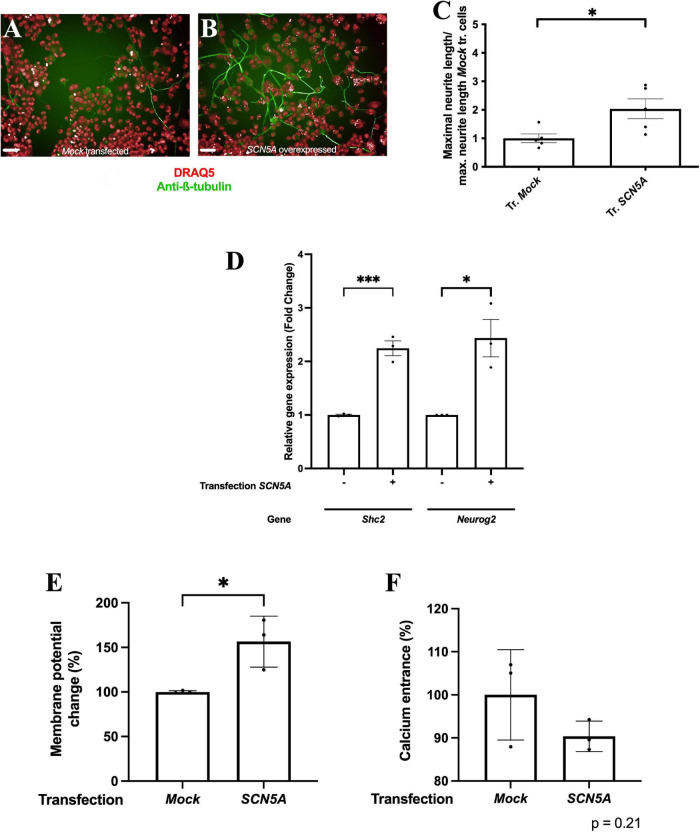
Overexpression of the gene encoding the Na_*V*_1.5 channel induces the acquisition of phenotypic features of DRG neurons. Representative images of **(A)** F11 cells transfected with an empty plasmid and **(B)** F11 cells transfected with the *SCN5A* gene. Images are representative of five independent assays (*n* = 5) with six replicates per condition, 20X. Scale bar = 50 μm. **(C)** Mean neurite length of F11 cells transfected with *SCN5A* and with an empty plasmid. Values shown for **(C)** are the means ± SEM of five independent assays (*n* = 5) with six replicates per measurement. *p < 0.05 (Student’s *t*-test). **(D)** Comparative quantification of *Shc2* and *Neurog2* gene expression by RT–qPCR in F11 cells transfected with an empty plasmid and with an *SCN5A* gene-containing plasmid. Values shown are the means ± SD of three independent assays (*n* = 3) with three replicates per measurement. ^***^p < 0.001; *p < 0.05 (Student’s *t*-test). **(E)** Membrane potential change measured with FluoVolt elicited by 30 mM KCl in F11 cells transfected with an empty plasmid and with an *SCN5A* gene-containing plasmid. Values shown are the means ± SD of three independent assays (*n* = 3) with four replicates per measurement. *p < 0.05 (Student’s *t-*test). **(F)** Intracellular calcium concentration increase elicited by 30 mM KCl in F11 cells transfected with an empty plasmid and with an *SCN5A* gene-containing plasmid. Values shown are the means ± SD of three independent assays (*n* = 3) with four replicates per measurement.

Our results indicated that the *Scn5a* gene may be involved in F11 cell differentiation. In addition to its role in the previously observed increase in intracellular sodium concentration after differentiation (Veerman et al., 2015), we found that the increase in intracellular sodium concentrations caused by overexpression of the gene encoding the Na_*V*_1.5 voltage-gated sodium channel (Supplementary Figure 4A) induced an increase in neurite length compared to non-transfected cells (Figures 3A–C) (*p* < 0.05, Student’s *t-*test). We observed that this acquisition of neuronal features was also accompanied by an increase in the expression of two genes encoding proteins involved in neuronal differentiation and neuritogenesis: *Shc2* and *Neurog2* (*p* < 0.001 for Shc2, *p* < 0.05 for *Neurog2*, Student’s *t-*test) (Figure 3D).

Overexpression of the Na_*V*_1.5 sodium channel also induced an increase in F11 cell depolarization in response to changes in extracellular medium KCl concentrations (Figure 3E) (*p* < 0.01, Student’s *t*-test) but not a significant increase in cytoplasmic calcium entrance compared to F11 cells transfected with an empty plasmid (Figure 3F) (*p* = 0.21, Student’s *t-*test). These data also support the involvement of sodium transients in neuronal differentiation.

### Pharmacological Inhibition of the Voltage-Gated Sodium Channel Reduces the Acquisition of Neuronal Phenotypic Features in Response to Differentiation of F11 Cells

**FIGURE 4:**
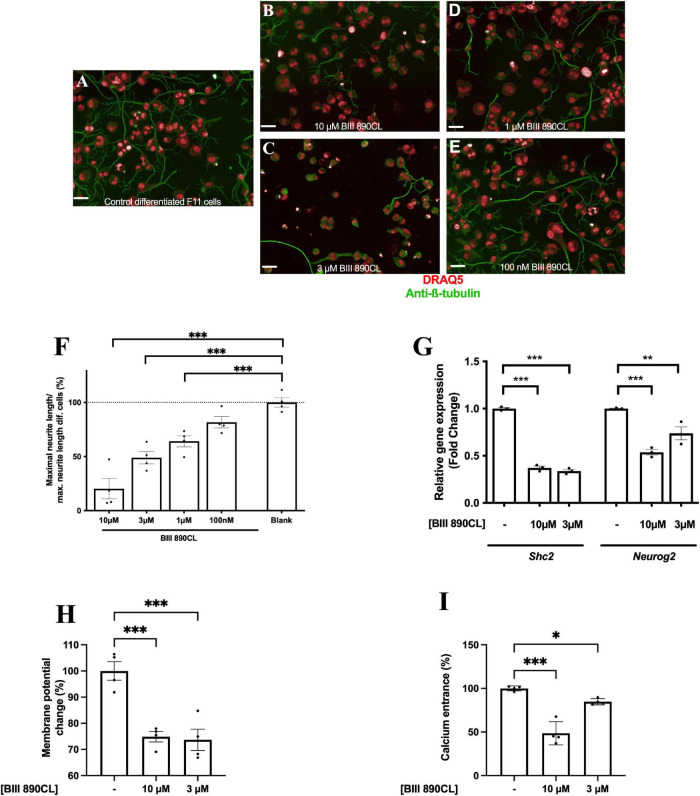
Pharmacologic inhibition of Na_*V*_1.5 channels induces a reduction in the acquisition of features of DRG neurons after differentiation. Representative images of **(A)** control differentiated F11 cells and F11 cells differentiated under exposure to **(B)** 10 μM BIII 890CL, **(C)** 3 μM BIII 890CL, **(D)** 1 μM BIII 890CL and **(E)** 100 nM BIII 890CL. Images are representative of four independent assays (*n* = 4) with four replicates per condition, 20X. Scale bar = 50 μm. **(F)** Maximal neurite length after 3 days of differentiation of F11 cells exposed to serial concentrations of BIII 890CL and control differentiated F11 cells. Values shown for **(F)** are the means ± SEM of four assays (*n* = 4) with four replicates per measurement. ^***^p < 0.001 (ANOVA test followed by Dunett’s *post hoc* analysis). **(G)** Comparative quantification of the expression of the *Shc2* and *Neurog2* genes by RT–qPCR in differentiated F11 cells in the absence and presence of 10 and 3 μM BIII 890CL. Values shown are the means ± SD of three independent assays (*n* = 3) with three replicates per measurement. ^**^p < 0.01; ^***^p < 0.001 (ANOVA test followed by Dunett’s *post hoc* analysis). **(H)** Membrane potential change measured with FluoVolt elicited by 30 mM KCl in differentiated F11 cells in the absence and presence of 10 and 3 μM BIII 890CL. Values shown are the means ± SD of four independent assays (*n* = 4) with three replicates per measurement. ^***^p < 0.001 (ANOVA test followed by Dunett’s *post hoc* analysis). **(I)** Intracellular calcium concentration increase elicited by 30 mM KCl in differentiated F11 cells in the absence and presence of 10 and 3 μM BIII 890CL. Values shown are the means ± SD of four independent assays (*n* = 4) with three replicates per measurement. *p < 0.05; ^***^p < 0.001 (ANOVA test followed by Dunett’s *post hoc* analysis).

To confirm the role of intracellular sodium concentration in F11 cell differentiation, we exposed differentiating F11 cells to the voltage-gated sodium channel inhibitor BIII 890CL, which reduces intracellular sodium concentration in a dose-dependent manner (Supplementary Figure 4B). We observed that exposure to BIII 890CL reduced the neurite length of differentiated F11 cells in a dose-dependent manner compared to that of control differentiated cells (Figures 4A–E) (*p* < 0.001 for 10 μM, 3 μM and 1 μM; ANOVA followed by Dunett’s *post hoc* analysis) (Figure 4F). Additionally, we observed that BIII 890CL exposure reduced expression of the *Shc2* and *Neurog2* genes (both encoding proteins involved in neuronal differentiation) in differentiated F11 cells (Figure 4G) (*p* < 0.001 for *Shc2* and *p* < 0.01 for *Neurog2*; ANOVA followed by Dunett’s *post hoc* analysis).

Blockade of sodium transients using BIII 890CL also reduced the excitability of differentiated F11 cells in a dose-dependent manner as measured either by the changes in membrane potential (Figure 4H) (*p* < 0.001; ANOVA followed by Dunett’s *post hoc* analysis) or the increase in calcium transients (Figure 4I) (*p* < 0.001 for 10 μM BIII 890CL and *p* < 0.05 for 3 μM BIII 890CL; ANOVA followed by Dunett’s *post hoc* analysis) induced by KCl.

To confirm the specificity of these effects, we employed BIII-55CL, a structurally similar analog of BIII 890CL with a 1000-fold lower potency (Carter et al., 2000), and we observed that BIII-55CL did not modify the phenotype of differentiated F11 cells as BIII 890CL did. Only a small reduction in neurite length at 10 μM BIII-55CL, the highest concentration assayed (Supplementary Figure 5) (*p* < 0.05; ANOVA followed by Dunett’s *post hoc* analysis), was observed. These data confirmed the role of sodium transients in F11 cell differentiation. Also, we observed that the exposition of F11 cells to 100 nM tetrodotoxin nor to 200 nM PF-01247324 did not induce a significant effect in the process of F11 cells differentiation (Supplementary Figure 6).

## Discussion

This work aimed to shed light on the F11 cell differentiation process by employing a combination of transcriptomic and functional approaches to deconvolute the molecular mechanisms of F11 cell differentiation to DRG neurons and the mechanisms involved in neuritogenesis.

Our major finding was the demonstration of Na_*V*_1.5 sodium channel involvement in the differentiation process of immortalized F11 cells into DRG neurons. Although it has been previously described that sodium participates in axonal guidance in DRG neurons (Yamane et al., 2017), to our knowledge, this work is the first in which sodium transient involvement in peripheral neuronal differentiation has been described.

We observed that differentiation of F11 cells induced the outgrowth of neurites, as previously shown by different authors (Ghil et al., 2000; Wieringa et al., 2012). The neurite length increase was accompanied by an increased membrane depolarization induced by KCl and by a rise in cytoplasmic sodium concentration in response to changes in extracellular medium NaCl concentration.

In previous work (Martínez et al., 2019), we observed that F11 cell differentiation induced an increase in intracellular calcium concentration in response to KCl through voltage-gated calcium channels. Furthermore, we observed that F11 cell differentiation elicited a reduction in SOCE, indicating that the entrance of calcium induced by KCl was due to an increase in the direct entrance of calcium from the extracellular medium and not related to the release of calcium stores from the endoplasmic reticulum. This result agreed with the observed increase in the transcription of genes encoding voltage-gated calcium channels and the reduction in the transcription of genes encoding proteins involved in SOCE, especially genes encoding transient receptor potential canonical (TRPC) channels (Supplementary Table 1).

The observed increase in neurite length and in the excitability of F11 cells in response to differentiation led us to investigate which genes were involved in this process by analyzing the F11 cell transcriptome before and after differentiation. Differentiation elicited changes in the expression of 13,880 murine genes and 11,291 rat genes. We focused our study on the expression of genes involved in the acquisition of neuronal features. In particular, we observed an increase in the transcription of genes involved in pathways related to the regulation of membrane potential, such as *Scn5a*, and to DRG neuron development and neuritogenesis, such as *Shc2* and *Neurog2*. According to the decay in growth rate after differentiation described by Wieringa et al. (2012), we observed that differentiation also induced a reduction in the transcription of genes encoding proteins related to pathways implicated in cell division.

The overexpression of genes encoding voltage-gated sodium channels and the observed intracellular sodium concentration increase after differentiation led us to hypothesize that sodium currents may play a major role in differentiation. The Na_*V*_1.5 channel was first cloned in the heart and was previously considered a cardiac sodium channel (Catterall, 1986); however, it was subsequently described in neural tissues, including DRG neurons (Wang et al., 2018). Furthermore, it was reported that expression of this gene was highly increased during neuronal differentiation of stem cells (Jang et al., 2010; Singh et al., 2017). For this reason, we evaluated the expression of neuronal phenotypic features in response to overexpression of the Na_*V*_1.5 channel (encoded by *Scn5a*). We observed that overexpression of the Na_*V*_1.5 channel in non-differentiated F11 cells elicited a twofold increase in the maximal neurite length compared to control non-transfected cells, suggesting participation of this sodium channel in neurite growth. Furthermore, we observed that Na_*V*_1.5 channel overexpression induced increased F11 cell depolarization when the cells were exposed to changes in extracellular medium KCl concentration. Moreover, after Na_*V*_1.5 channel overexpression, there was a significant increase in the expression of genes involved in DRG neuron differentiation, such as *Shc2a* and *Neurog2*. The *Shc2* gene encodes ShcB, a docking protein that participates in TrkA-mediated signaling during DRG neuron development that is crucial for DRG neuron survival (Sakai et al., 2000). *Neurog2* encodes neurogenin 2, a transcription factor whose expression is highly increased during the differentiation of sensory neurons (Ma et al., 1999). The acquisition of a DRG neuron phenotype and this observed increase in the transcription of genes related to DRG neuron differentiation after overexpression of Na_*V*_1.5 in F11 cells is in agreement with the finding that sodium influx activates the CREB transcription factor, leading to enhanced neurite formation and axonal elongation (Zou et al., 2017).

Intriguingly, despite the observed increase in neurite length and excitability in Na_*V*_1.5-overexpressing F11 cells, no significant changes occurred with respect to the entrance of calcium into the cytoplasm in response to extracellular medium KCl concentration changes. Similar results were reported by Ryglewski et al. (2014) in Drosophila motoneurons, who described that the first ion currents that appeared during neuronal differentiation were voltage-gated calcium currents, followed by voltage-gated sodium currents connected to dendrite development (Ryglewski et al., 2014). We infer that this increase in voltage-gated calcium channel expression during development is independent of any other voltage-gated ion current, although other neuronal features, such as neurite growth, are related to sodium entrance into the cytoplasm. Furthermore, our results demonstrated that the increase in calcium channel expression after differentiation was not mediated by sodium transients.

We also examined the effect of blocking sodium channels during differentiation by exposing differentiating F11 cells to BIII 890CL, a selective voltage-gated sodium channel blocker that inhibits action potentials elicited by TTX-insensitive voltage-gated sodium channels, such as Na_*V*_1.5 (Carter et al., 2000; Krause et al., 2000). We observed reduced neurite outgrowth together with decreased depolarization and calcium transients in response to KCl that was concentration-dependent. These findings suggest that although the increase in sodium transients observed during neuronal differentiation did not induce an increase in the expression of voltage-gated calcium channels *per se*, the inhibition of sodium transients induced a reduction in voltage-gated calcium currents. This effect may be because BIII 890CL is a non-selective sodium channel blocker, so the reduction in sodium entrance due to the compound could be more pronounced than sodium entrance enhancement by overexpressing the Na_*V*_1.5 channel.

These effects of BIII 890CL during differentiation resembled the response of non-differentiated F11 cells and concurred with the observed decrease in the expression of *Shc2* and *Neurog2* genes involved in the differentiation of DRG neurons, indicating that pharmacologic inhibition of the Na_*V*_1.5 channel hampers the differentiation of F11 cells. To confirm the specificity of this effect, we also employed BIII-55CL, a structural analog of BIII 890CL that possesses a 1,000-fold decreased potency for sodium channels (Carter et al., 2000), observing that this compound only elicited a small reduction in neurite length at the highest concentration employed and did not modify cell excitability. We exposed F11 cells during differentiation to tetrodotoxin, that inhibits voltage-gated sodium channels but not Na_*V*_1.5 sodium channel (Ogata and Ohishi, 2002), and to PF-01247324, that inhibits Na_*V*_1.8 voltage-gated sodium channel (Payne et al., 2015). We did not observe a significant effect of both molecules in neurite length, confirming the specificity of the role of Na_*V*_1.5 in the differentiation of F11 cells.

The observed induction of neuronal phenotypic feature acquisition after overexpression of a sodium channel and the hampering of F11 cell differentiation by blocking sodium channels indicate a key role of sodium transients in DRG neuron differentiation and an interplay between sodium and calcium signaling. Indeed, sodium acts as a second messenger, inducing calcium release from the mitochondria (Hernansanz-Agustín et al., 2020).

In summary, using functional and transcriptomic assays, we elucidated the molecular mechanisms related to the differentiation process in a sensory neuron-derived cell line and observed that the Na_*V*_1.5 voltage-gated sodium channel plays an essential role in neuronal differentiation and neuritogenesis. Overexpression of the Na_*V*_1.5 voltage-gated sodium channel enhanced neuronal feature acquisition in DRG neuron-like cells, while pharmacological blockade of the channel prevented cell line differentiation in a concentration-dependent manner. These results contribute to the understanding of the molecular mechanisms of neuritogenesis and neuronal differentiation in F11 cells and shed light on novel therapeutic approaches for enhancing neuronal plasticity after peripheral nerve injury.

## Data Availability Statement

The original contributions presented in the study are publicly available. These data can be found here: https://www.ncbi.nlm.nih.gov/sra/PRJNA808958.

## Author Contributions

AM: investigation, methodology, visualization, and writing—original draft. JBr: conceptualization, supervision, writing and original draft, and formal analysis. ED: conceptualization, visualization, and writing—review and editing. MV: investigation, formal analysis, and writing—review and editing. CA: data curation, formal analysis, and methodology. RC: data curation, formal analysis, and visualization. XM: resources. MM: conceptualization and writing—review and editing. JBu: supervision and writing—review and editing. ÁC: supervision. ML: supervision, conceptualization, and writing—review and editing. All authors discussed the results and commented on the manuscript.

## Conflict of Interest

WeLab Barcelona provided support in the form of salaries for XM, MM, and JBu, but did not have any additional role in the study design, data collection and analysis, decision to publish, or preparation of the manuscript. The remaining authors declare that the research was conducted in the absence of any commercial or financial relationships that could be construed as a potential conflict of interest.

## Publisher’s Note

All claims expressed in this article are solely those of the authors and do not necessarily represent those of their affiliated organizations, or those of the publisher, the editors and the reviewers. Any product that may be evaluated in this article, or claim that may be made by its manufacturer, is not guaranteed or endorsed by the publisher.
