# Supplementary material for: Identification of Sodium Transients Through NaV1.5 Channels as Regulators of Differentiation in Immortalized Dorsal Root Ganglia Neurons
Source: Front Cell Neurosci. 2022 Apr 6;16:816325. doi: 10.3389/fncel.2022.816325 (PMC9018981; doi:10.3389/fncel.2022.816325)
Supplement: Supplementary file 2 [file Data_Sheet_1.DOCX]

Supplementary Material

# Supplementary Data

**Supplementary video. Differentiation of F11 cells elicits an increase in neurite length.** Time-lapse video depicting the differentiation of F11 cells for 72 hours. Video represents one field of one well and is representative of two independent assays (n=2) with eleven replicates per condition, 10X.

# Supplementary Figures and Tables

## Supplementary Figures

**
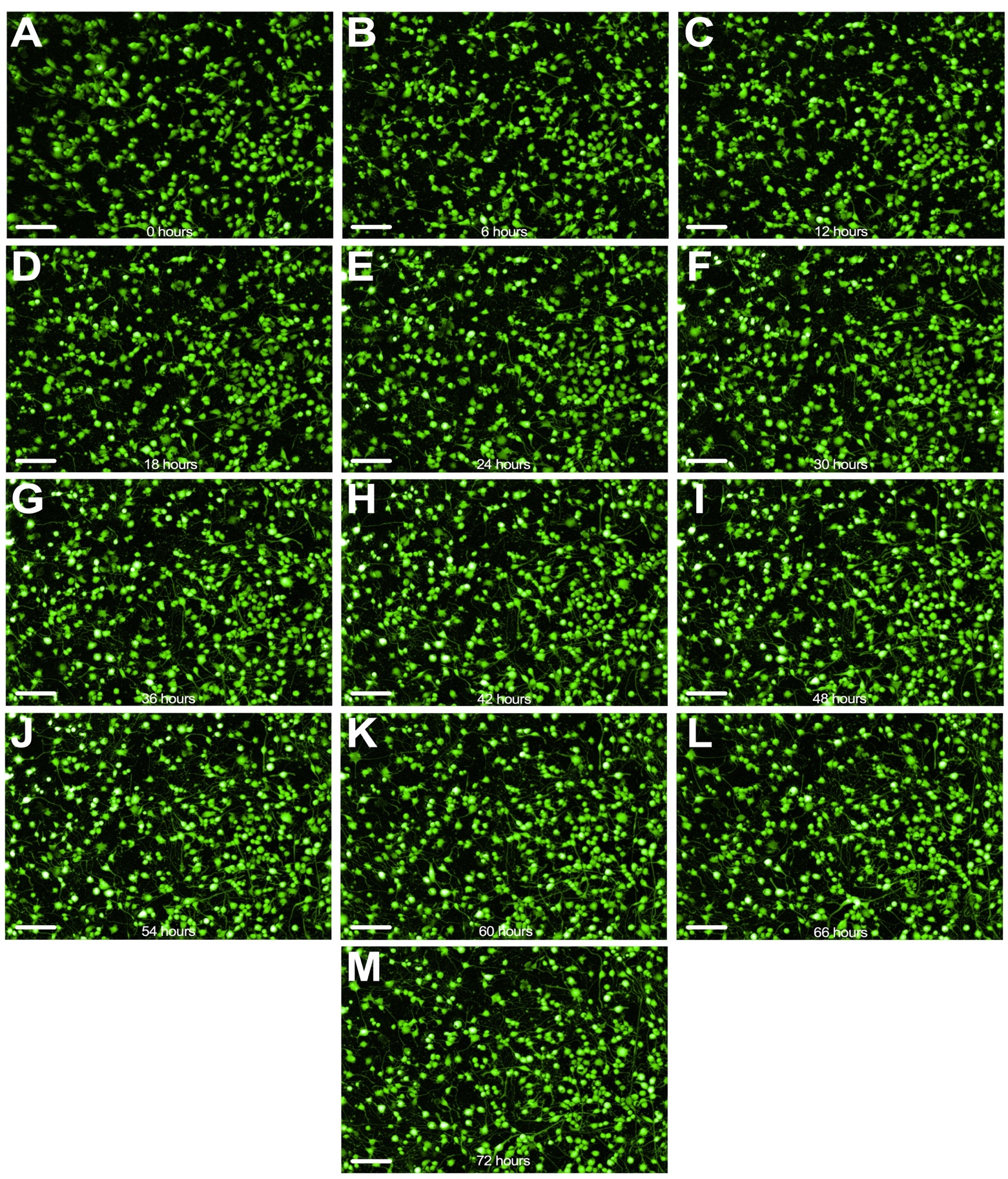
**

**Figure S1. Differentiation of F11 cells induces an increase in neurite length with a continuous increase beginning 12 hours after the addition of differentiation medium.** Representative images of F11 cells (A) 0, (B) 6, (C) 12, (D) 18, (E) 24, (F) 30, (G) 36, (H) 42, (I) 48, (J) 54, (K) 60, (L) 66 and (M) 72 hours after the addition of differentiation medium. Images are representative of two independent assays (n=2) with eleven replicates per condition, 10X. Scale bar = 100 µm.

**Figure S2. Differentiation of F11 cells elicited an increase in KCl-induced excitability.** Changes in membrane potential induced by 30 mM KCl in differentiated and in nondifferentiated F11 cells. Values of one representative experiment of three independent experiments (n=3).


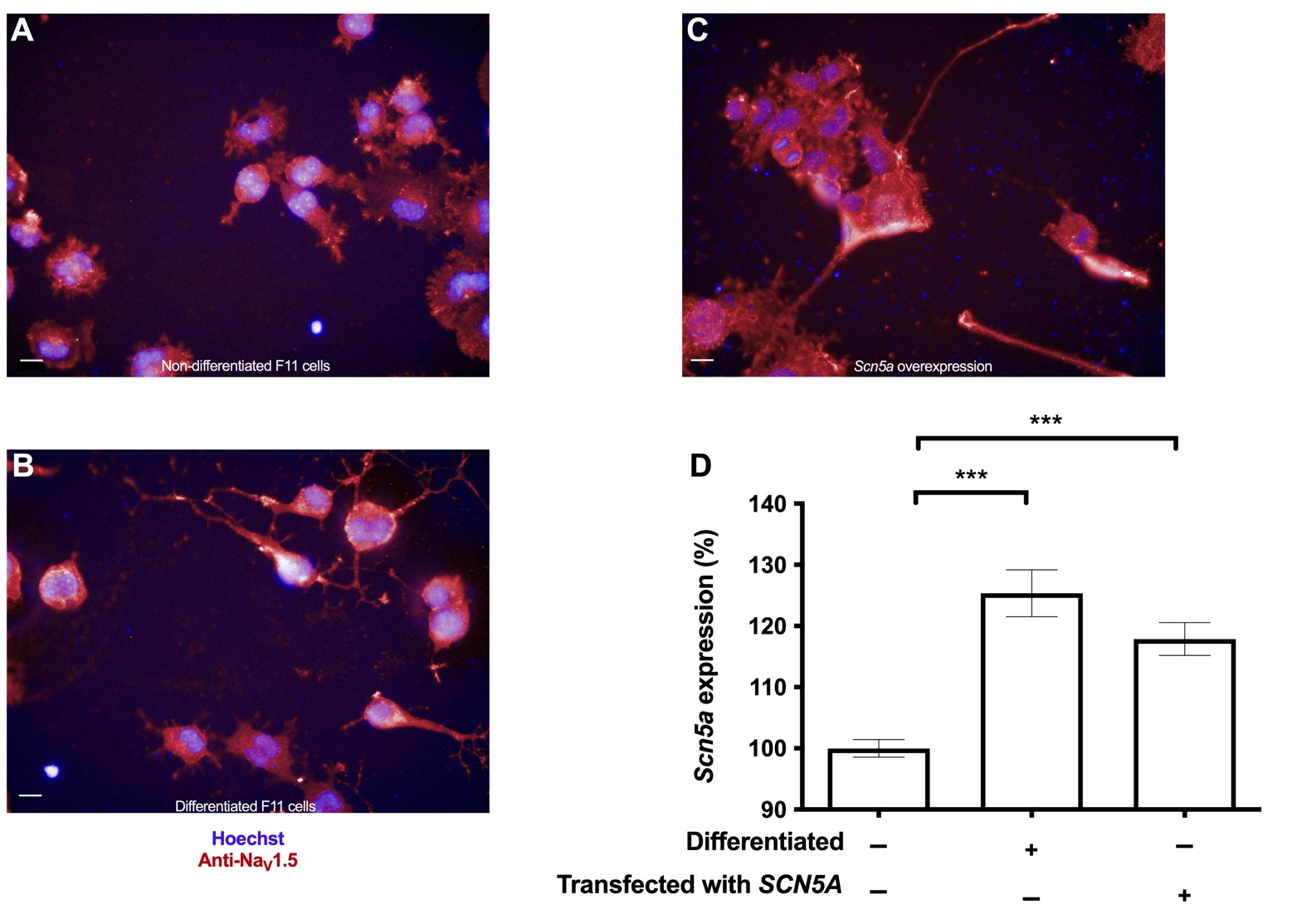


**Figure S3. Differentiation of F11 cells increases the expression of Na_V_1.5 voltage-gated sodium channels.** Representative images of (**A**) nondifferentiated F11 cells, (**B**) differentiated F11 cells and (**C**) F11 cells in which the Na_V_1.5 channel was overexpressed. Images are representative of two independent assays with three replicates per condition, 40X. Scale bar = 20 µm. (**D**) Na_V_1.5 channel expression in nondifferentiated F11 cells, differentiated F11 cells and F11 cells in which the *SCN5A* gene was overexpressed. Values shown for **D** are the means ± SEM of two independent assays using at least three replicates per measurement. ***p < 0.001 (ANOVA followed by Dunett's post hoc analysis).


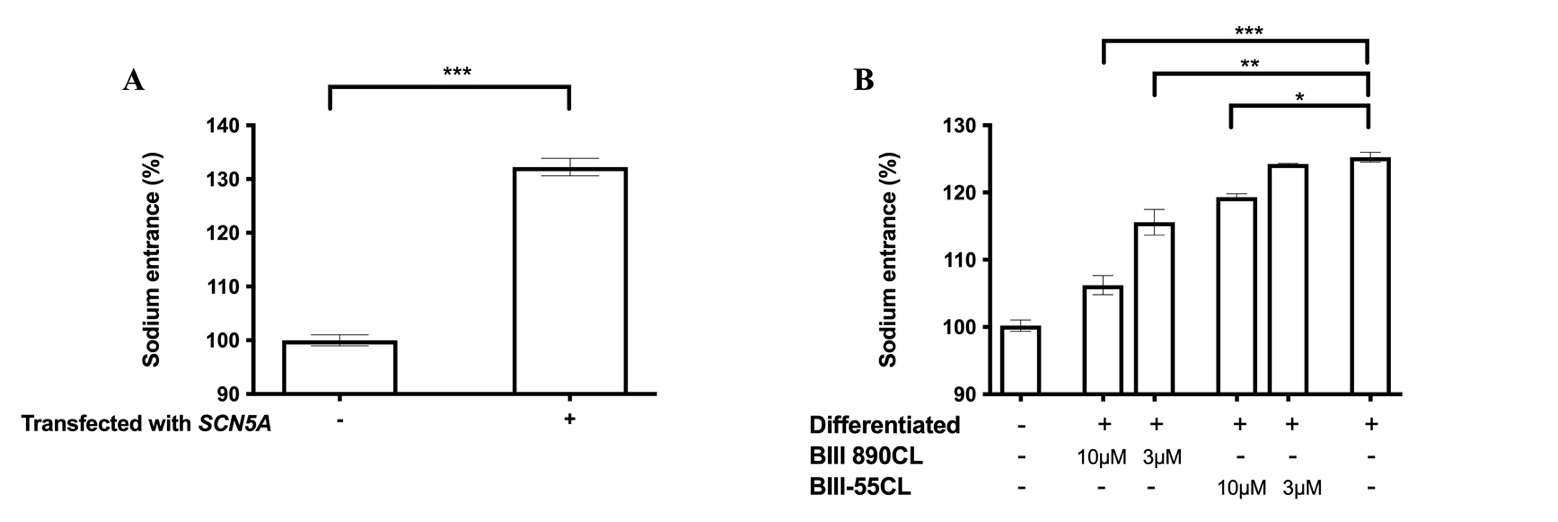


**Figure S4. Transfection of the *SCN5A* gene increases intracellular sodium concentrations in F11 cells, and Na_V_1.5 channel blockade reduces intracellular sodium concentrations in F11 cells.** (**A**) Intracellular sodium concentration in F11 cells transfected with an empty plasmid and in F11 cells in which the Na_V_1.5 channel was overexpressed. ***p < 0.001 (Student's *t* test). (**B**) Intracellular sodium concentration in nondifferentiated F11 cells, differentiated F11 cells exposed to different concentrations of BIII 890CL and BIII-55CL and control differentiated F11 cells. Values shown are the means ± SEM of one representative assay of three independent assays (n=3) with three replicates per measurement. * p < 0.05; ** p < 0.01; *** p < 0.001 (ANOVA followed by Dunett's post hoc analysis).


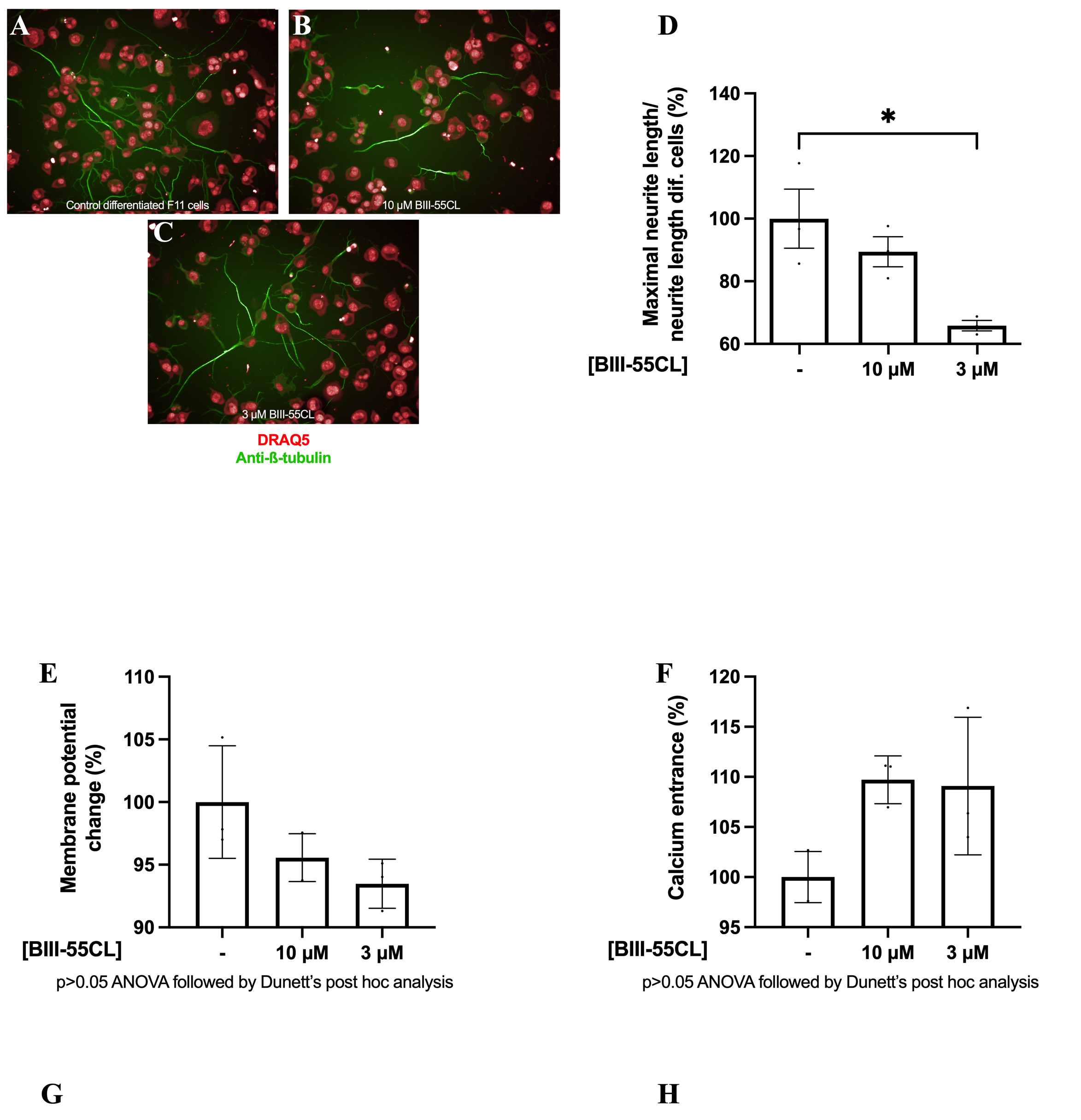


**Figure S5. BIII-55CL, a structural analogue of BIII 890CL with less potency, induced fewer changes in the phenotype of differentiated F11 cells.** Representative images of (**A**) control differentiated F11 cells and F11 cells differentiated under exposure to (**B**) 10 µM BIII-55CL and (**C**) 3 µM BIII-55CL. Images are representative of three independent assays (n=3) with three replicates per condition, 20X. (**D**) Maximal neurite length after three days of differentiation with and without exposure to serial concentrations of BIII-55CL. Values shown for **D** are the means ± SEM of one representative assay of three independent assays (n=3) with three replicates per measurement. *p < 0.05 (ANOVA followed by Dunett's post hoc analysis). **(E)** Membrane potential increase measured with FluoVolt elicited by 30 mM KCl in differentiated F11 cells in the absence and presence of 10 and 3 µM BIII-55CL. Values shown are the means ± SD of one representative assay of three independent assays (n=3) with three replicates per measurement. **(F)** Intracellular calcium concentration increase elicited by 30 mM KCl in differentiated F11 cells in the absence and presence of 10 and 3 µM BIII-55CL.

**
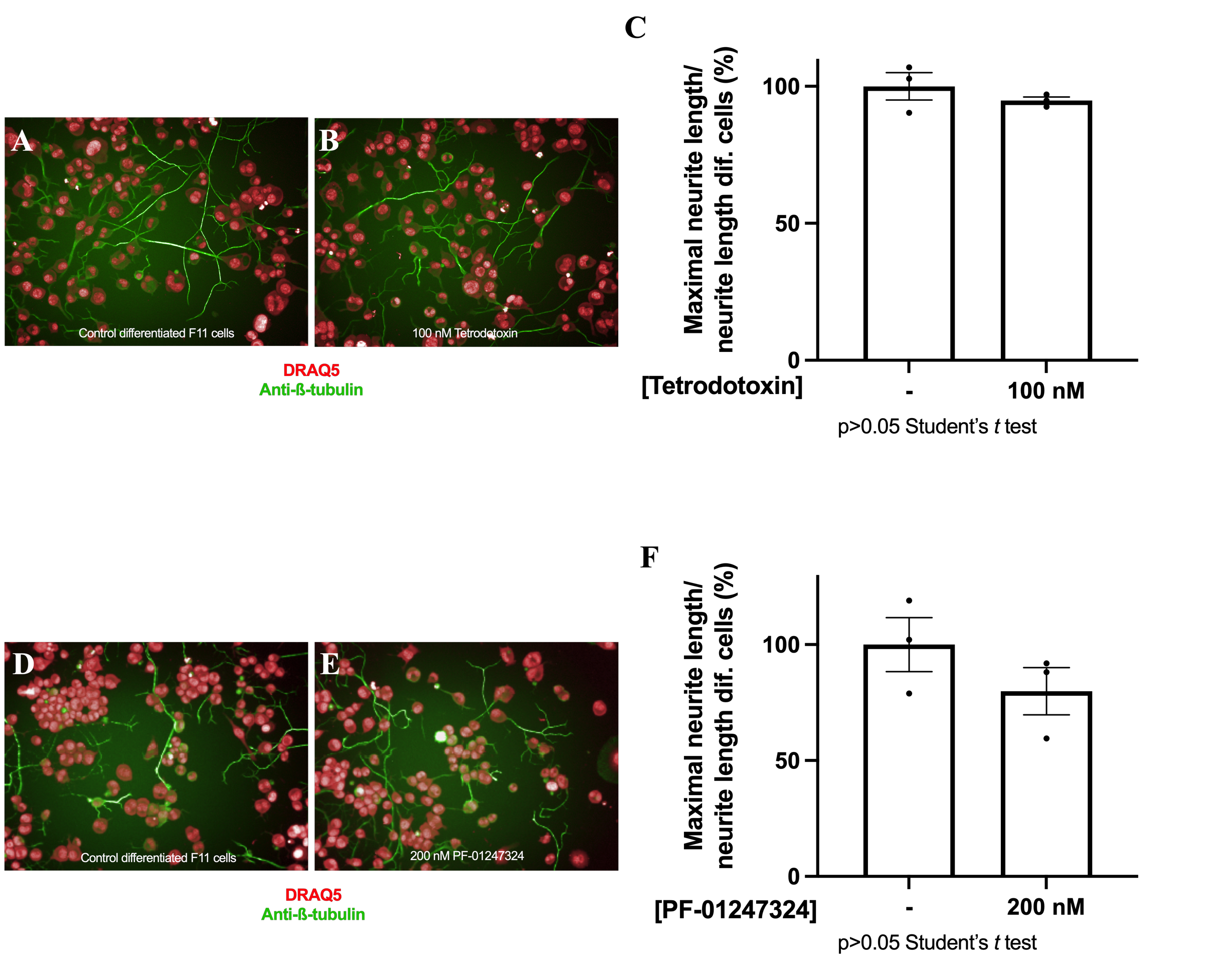
**

**Figure S6. Tetrodotoxin (TTX), a sodium channel inhibitor with less effect on Na_V_1.5 than in other sodium channels, and NaV1.8 sodium channel blocker PF-01247324 induced small changes in the phenotype of differentiated F11 cells.** Representative images of (**A**) control differentiated F11 cells and (**B**) F11 cells differentiated under exposure to 100 nM TTX. Images are representative of three independent assays (n=3) with three replicates per condition, 20X. (**C**) Maximal neurite length after three days of differentiation with and without exposure to 100 nM tetrodotoxin (TTX). Values shown for **C** are the means ± SEM of three independent assays (n=3) with four replicates per measurement. Representative images of (**D**) control differentiated F11 cells and (**E**) F11 cells differentiated under exposure to 200 nM PF-01247324. Images are representative of three independent assays (n=3) with three replicates per condition, 20X. (**F**) Maximal neurite length after three days of differentiation with and without exposure to 200 nM PF-01247324. Values shown for **F** are the means ± SEM of three independent assays (n=3) with four replicates per measurement.

## Supplementary Tables

**Table S1.** Differences in the expression of genes encoding proteins related to calcium permeability and SOCE in F11 cells before and after differentiation quantified by RNA-Seq. Data are the mean of three independent experiments using different samples.

| **Symbol** | **Mouse** | | **Rat** | |
| --- | --- | --- | --- | --- |
|  | **Position** | **Expr. Log Ratio** | **Position** | **Expr. Log Ratio** |
| *Orai1* | 1433/13880 | 0.911 | 960/11291 | 1.057 |
| *Stim1* | 4457/13880 | 0.276 | 3444/11291 | 0.342 |
| *Trpc1* | 2098/13880 | 0.692 | 1596/11291 | 0.760 |
| *Trpc3* | 13029/13880 | -0.922 | 10395/11291 | -0.751 |
| *Trpc5* | 13372/13880 | -1.201 | 11002/11291 | -1.343 |
| *Cacna1 g* | 696/13880 | 1.359 | 496/11291 | 1.476 |
| *Cacna2d3* | 1615/13880 | 0.845 | 1347/11291 | 0.852 |
| *Cacna2d2* | 1778/13880 | 0.786 | 1431/11291 | 0.818 |
| *Cacna1e* | 4766/13880 | 0.239 | 4653/11291 | 0.173 |
| *Cacna1 h* | 4998/13880 | 0.212 | 3852/11291 | 0.279 |
| *Cacna1a* | 5618/13880 | 0.143 | 3878/11291 | 0.275 |
| *Cacna1b* | 5691/13880 | 0.137 | 4617/11291 | 0.178 |
| *Cacna2d1* | 6324/13880 | 0.072 | 5650/11291 | 0.058 |
| *Cacna1i* | 9847/13880 | -0.267 | 7975/11291 | 0.216 |
